# Exercise-induced angiogenesis is attenuated by captopril but maintained under perindopril treatment in hypertensive rats

**DOI:** 10.3389/fphys.2023.1147525

**Published:** 2023-05-22

**Authors:** Anderson G. Macedo, Danyelle S. Miotto, Lidieli P. Tardelli, Carlos F. Santos, Sandra L. Amaral

**Affiliations:** ^1^ Department of Physical Education, School of Sciences, São Paulo State University, Bauru, Brazil; ^2^ Joint Graduate Program in Physiological Sciences (PIPGCF), Federal University of São Carlos and São Paulo State University, São Carlos, Brazil; ^3^ Department of Biological Sciences, Bauru School of Dentistry, University of São Paulo (USP), Bauru, Brazil

**Keywords:** ACE inhibitors, vessel growth, aerobic training, tibialis anterior, VEGF, eNOS

## Abstract

Angiogenesis is an important exercise-induced response to improve blood flow and decrease vascular resistance in spontaneously hypertensive rats (SHR), but some antihypertensive drugs attenuate this effect. This study compared the effects of captopril and perindopril on exercise-induced cardiac and skeletal muscle angiogenesis. Forty-eight Wistar rats and 48 SHR underwent 60 days of aerobic training or were kept sedentary. During the last 45 days, rats were treated with captopril, perindopril or water (Control). Blood pressure (BP) measurements were taken and histological samples from the tibialis anterior (TA) and left ventricle (LV) muscles were analyzed for capillary density (CD) and vascular endothelial growth factor (VEGF), VEGF receptor-2 (VEGFR-2) and endothelial nitric oxide synthase (eNOS) protein level. Exercise increased vessel density in Wistar rats due to higher VEGFR-2 (+17%) and eNOS (+31%) protein level. Captopril and perindopril attenuated exercise-induced angiogenesis in Wistar rats, but the attenuation was small in the perindopril group, and this response was mediated by higher eNOS levels in the Per group compared to the Cap group. Exercise increased myocardial CD in Wistar rats in all groups and treatment did not attenuate it. Both exercise and pharmacological treatment reduced BP of SHR similarly. Rarefaction was found in TA of SHR compared to Wistar, due to lower levels of VEGF (−26%) and eNOS (−27%) and treatment did not avoid this response. Exercise prevented these reductions in control SHR. While rats treated with perindopril showed angiogenesis in the TA muscle after training, those rats treated with captopril showed attenuated angiogenesis (−18%). This response was also mediated by lower eNOS levels in Cap group compared with Per and control group. Myocardial CD was reduced in all sedentary hypertensive compared with Wistar and training restored the number of vessels compared with sedentary SHR. In conclusion, taken into account only the aspect of vessel growth, since both pharmacological treatments reduced BP in SHR, the result of the present study suggests that perindopril could be a drug of choice over captopril for hypertensive practitioners of aerobic physical exercises, especially considering that it does not attenuate angiogenesis induced by aerobic physical training in skeletal and cardiac muscles.

## 1 Introduction

Exercise induced angiogenesis is a physiological response that contributes to improve microcirculation perfusion, oxygen uptake and reduce vascular resistance, in addition to helping to control blood pressure (BP) in hypertensive individuals (Egginton, 2009; Rossoni et al., 2011; Hoier and Hellsten, 2014). The mechanisms underlying exercise induced angiogenesis in skeletal and cardiac muscle are not completely understood, but some authors have focused their attention on the vascular endothelial growth factor (VEGF) and nitric oxide (NO), which are the two more important factors involved in angiogenesis under physiological and pathological situations (Gavin et al., 2000; Milkiewicz et al., 2005; Amaral et al., 2008; Ferrara, 2009; Hoier et al., 2010; Gavin et al., 2015; Karimian et al., 2015).

Renin angiotensin system (RAS), besides its role in the regulation of BP and body fluids, is an important regulator of microvessel growth. Over the past 2 decades, studies from our group have shown that RAS has a crucial role in controlling skeletal muscle capillary density, mainly through modulation of VEGF levels (Amaral et al., 2001a; Amaral et al., 2001b; Amaral et al., 2001c; de Resende et al., 2008). Consequently, RAS inhibition, especially with captopril, lisinopril, losartan as well as genetic manipulations, has been shown to attenuate physiological or pathological angiogenesis (Gavin et al., 2000; Amaral et al., 2001a; Amaral et al., 2001b; de Resende et al., 2008). As angiotensin converting enzyme (ACE) inhibitors attenuate the exercise-induced angiogenesis, our group was concerned about the effects of the combination of ACE inhibitors, which are very effective in reducing BP, with aerobic physical training, since the increase in vessels is a specific and crucial response induced by training. Thus, physically active hypertensive patients may not benefit from this combination. Among all ACE inhibitors, captopril was the first to be released for use (in 1981) and has been prescribed to control BP, but a large study evaluating 43,356 patients with congestive heart failure showed that lisinopril, fosinopril and perindopril were more effective then captopril, which showed the lowest benefits in reducing the risk of mortality these patients (Pilote et al., 2008). In addition, it has been shown that captopril, a sulfhydryl ACE inhibitor, inhibits angiogenesis (Fabre et al., 1999; Gavin et al., 2000; Amaral et al., 2001b; Bellamy et al., 2010). In contrast, some studies have shown that perindopril, a non-sulfhydryl ACE inhibitor, does not impair skeletal muscle angiogenesis under ischemic conditions (Silvestre et al., 2002) or after aerobic exercise on a treadmill in female rats (Guo et al., 2010). Althogh promising, there are very few information about the effects of perindopril on the capillary growth after exercise training (Minami et al., 2007; Rouyer et al., 2007; Habouzit et al., 2009; Guo et al., 2010).

Therefore, the aim of the present study was to compare the effects of captopril and perindopril on aerobic exercise-induced angiogenesis of cardiac and skeletal muscles and to investigate whether VEGF and NO could modulate these responses in healthy and hypertensive rats. We hypothesized that rats treated with perindopril would not show an inhibition of angiogenesis compared to captopril in skeletal and cardiac muscle induced by aerobic physical training.

## 2 Methods

Forty-eight male Wistar rats (200–250 g) and 48 male spontaneously hypertensive rats (SHR) were obtained from animal breeding facility at São Paulo State University, UNESP (Botucatu, SP, Brazil) and Institute of Biomedical Sciences of University of Sao Paulo, USP (São Paulo, Brazil). Rats were allocated in collective cages (up to 5 animals per cage) at the Animal Facility at School of Sciences, UNESP, campus of Bauru (12 h dark-light cycle, 22°C). They received standard diet (NUVILAB^®^, Brazil) and water *ad libitum.* All methods were approved by The Committee for Ethical Use of Animals (CEUA) of the School of Sciences, UNESP-Sao Paulo State University, campus of Bauru, SP, Brazil (# 523/2014 vol 1–CEUA/FC).

### 2.1 Groups

Rats were separated into 12 groups with similar body weight (BW) and maximal physical capacity and, randomly assigned to the following groups: 1) Wistar sedentary control, daily treated with saline or water (WSctr n = 8); 2) Wistar sedentary, daily treated with captopril (WScap n = 8); 3) Wistar sedentary, daily treated with perindopril (WSper n = 8); 4) Wistar trained control, daily treated with saline or water (WTctr n = 8); 5) Wistar trained, daily treated with captopril (WTcap n = 8); 6) Wistar trained, daily treated with perindopril (WTper n = 8). 7) Spontaneously hypertensive sedentary control, daily treated with saline or water (SHRSctr n = 8); 8) Spontaneously hypertensive sedentary, daily treated with captopril (SHRScap n = 8); 9) Spontaneously hypertensive sedentary, daily treated with perindopril (SHRSper n = 8); 10) Spontaneously hypertensive trained control, daily treated with saline or water (SHRTctr n = 8); 11) Spontaneously hypertensive trained, daily treated with captopril (SHRTcap, n = 8); 12) Spontaneously hypertensive trained, daily treated with perindopril (SHRTper, n = 8).

### 2.2 Maximum physical capacity and aerobic exercise protocol

All rats were weighed, adapted, and selected according to their ability to walk/run on a treadmill adjusted for rats (Inbramed, Porto Alegre, RS, Brazil) for 5–10 days. The maximal capacity was evaluated through a progressive maximal capacity test (Tmax), following the protocol previously published (Barel et al., 2010), which consisted of increments of 5 m/min every 3 min, 0% grade until the rat stopped running spontaneously. Maximal capacity test was repeated on the 4th week, to adjust the training velocity (and keep intensity) and after the 8th week, to evaluate exercise effectiveness. Sedentary rats were adapted every 15 days to maintain their ability to walk and performed the Tmax together with trained rats. Aerobic physical training (T) was performed on a treadmill at 60% of their maximum physical capacity, 0% grade, 1 h per day, 5 days/week for 60 days (Barel et al., 2010).

### 2.3 Pharmacological treatment

All pharmacological treatments were performed during the last 45 days of the experimental protocol (at 9 a.m.). Groups 2, 5, 8 and 11 were daily treated with Captopril (Captopril^®^, Sanofi Medley Farmacêutica Ltda, Brazil, 25 mg/kg of BW, *i.p.*, dissolved in saline (Sattar et al., 1985). Groups 3, 6, 9 and 12 received daily administration of perindopril (Conversyl^®^, Laboratórios Servier do Brasil Ltda, Brazil, 3 mg/kg of BW, *via gavage*, dissolved water, (Minami et al., 2007)). Control groups (1, 4, 7 and 10) were randomly treated with saline (*i.p*.) or water (*via gavage*), using the same volume that was used to dissolve captopril or perindopril.

### 2.4 Cardiovascular parameters

On the last day of pharmacological treatment, all rats underwent a carotid artery catheterization under anesthesia (xylazine hydrochloride, 10 mg/kg and ketamine hydrochloride, 50 mg/kg, CEVA Sante Animale, Paulínea, SP, Brazil) as published (Herrera et al., 2016). After 24 h of recovery, the external part of the catheter was connected to the recording system. Blood pressure (BP) was continuously recorded for at least 1 h (2,000 Hz of frequency), in a quiet room, using a pressure transducer (DPT100, Utah Medical Products Inc., Midvale, UT, United States), which sent the signal to an amplifier (Quad Bridge Amp, ADInstruments, Colorado Springs, CO, United States) and then to an acquisition board (Powerlab 4/35, ADInstruments, New South Wales, Australia), as published (Duchatsch et al., 2018). A computer software (Labchart pro v7.0, ADInstruments, New South Wales, Australia) derived pulsatile BP recordings and calculated mean blood pressure (MBP), systolic blood pressure (SBP), diastolic blood pressure (DBP) and heart rate (HR).

### 2.5 Tissue harvesting

After hemodynamic measurements, all rats were euthanized by an overdose of anesthesia (160 mg/kg of ketamine hydrochloride and 20 mg/kg of xylasine hydrochloride, CEVA Sante Animale, Paulínea, SP, Brazil). Thereafter, tibialis anterior muscle (TA) and myocardium were harvested, weighed, and cleaned. Part of the muscles were surrounded by neutral talc, covered with Optimal Cutting Temperature (OCT -*TISSUE-TEK* ^®^—Sakura Finetek, Torrance, CA, United States), inserted into a microcentrifuge tube and immediately dipped into liquid nitrogen for histological processing. Another part of the muscles was inserted into a microcentrifuge tube and immediately dipped into liquid nitrogen for protein analysis. After freezing, muscle samples were stored at −80°C for further analyses. Tibia bone length was measured for muscle weight normalization.

### 2.6 Morphometric evaluations

All muscle samples was placed into the cryostat (−20°C, Leica^®^ type CM 1850 Nussloch, Germany) and 10 µm transverse slices were performed. Next, all slices were mounted on glass slides and stained with hematoxylin-eosin (HE). Six microscopic fields (137,280 µm^2^) were randomly chosen from different areas of the transverse sections as already published (Herrera et al., 2016). A Controller program (Olympus micro DP70) digitized all images with Olympus BX50 microscope, with an objective of ×40. Digitized images were analyzed off-line for determination of capillary-to-fibers ratio (C:F Ratio) and capillary density (CD). The CD was determined by the number of capillaries identified/mm^2^ and C:F Ratio was obtained by the ratio between capillaries and fibers numbers using an Image.pro Plus 6.2 program. An average of 4-6 pictures was analyzed.

### 2.7 Westerns blotting procedures

TA muscle and myocardium were homogenized in RIPA buffer (Cell Signaling Technology^®^, Danvers, Massachusetts, United States), with 0.1% protease inhibitor cocktail (PIC, Sigma Aldrich, St. Louis, MO, United States) and 1% of phenylmethylsulfonyl fluoride (PMSF, Sigma Aldrich, St. Louis, MO, United States), using a Polytron homogenizer. Samples were centrifuged at 11,752.4 g per 5 min and then the supernatant was collected and stored at −20°C for future analysis. Bradford assay kit was used to determine protein concentration (Bio-Rad Kit, Protein Assay Standart II, Hercules, CA, United States) and absorbance values were determined using a spectrophotometric plate reader (BMG labtech, Spectro Star Nano, Ortenberg, Baden-Württemberg, Germany), as previously published (Macedo et al., 2014; Macedo et al., 2016). In summary, 30–50 µg of protein were separated using 8% and 10% denaturing polyacrylamide gel with running buffer for 60 min (190 mM glycine, 25 mM Tris, 0.1% SDS pH 8.3). These gels were then transferred to a nitrocellulose membrane using a transfer buffer (190 mM de glycine, 25 mM de Tris, 20% de methanol, pH 8.3) for 90 min. The equal protein loading was confirmed using a 0.5% *Ponceau* staining. Membranes were blocked with bovine serum albumin (BSA, 1%) in Tris-buffered saline with Tween-20 (TBS-T) for 120s. SNAPi.d.^®^ 2.0 Protein Detection system (Millipore, Darmstadt, Hessen, Germany) was used as previously published (Macedo et al., 2014). The membranes were incubated with primary antibodies for 10 min, prepared in 3% BSA: anti-human VEGF (anti-mouse, BD Pharmingen, # 55439, 1:1,000), anti-human eNOS (anti-mouse, BD Pharmingen, # 610297, 1:500), anti-VEGFR-2 (anti-rabbit, Cell Signaling, # 9698, 1:500) and anti-GAPDH (anti-rabbit, Santa Cruz, sc-25778, 1:1,000). The membranes were washed and incubated in the appropriate horseradish peroxidase-conjugated secondary antibody (anti-mouse or anti-rabbit according to each primary antibody source). Signals were detected by enhanced chemiluminescence (Super signal ®West Pico, Pierce), captured on radiographic film, and bands of interest were analyzed using scanning densitometry program (Scion Image, Corporation, Beta 4.02). Raw densitometry data for each target protein signal were normalized to the Glyceraldehyde-3-phosphate dehydrogenase (GAPDH), protein signal from the same sample. Group data are expressed as a percentage relative to the control group.

### 2.8 Statistical analysis

All values are presented as mean ± standard error of mean (SEM). The Normality of data were verified by Shapiro-Wilk. Two-way analysis of variance (ANOVA) was used, and significant differences were further investigated using a Tukey post-hoc test (*p* < 0.05).

## 3 Results

Table 1S (Supplementary Material) shows delta Tmax, final body weight, TA and left ventricle muscle mass, normalized by the tibia bone length and values of the SBP, MBP and DBP of the normotensive animals. All normotensive groups increased similarly their body weight along the protocol. Delta Tmax, represented by the delta values between the 3rd and 1st Tmax showed that T significantly increased the physical capacity of the normotensive groups, compared with their respective sedentary groups. Supplementary Table S1 (Supplementary Material) also shows that pharmacological treatment did not alter the animal´s physical capacity. All normotensive groups presented similar TA and heart muscle mass. All sedentary Wistar groups presented similar SBP, DBP and MBP and training or treatment did not significantly change these values (Supplementary Table S1).

Supplementary Table S2 (Supplementary Material) shows delta Tmax, final body weight, TA and left ventricle muscle mass, normalized by the tibia bone length and values of the SBP, MBP and DBP of the SHR. All hypertensive groups increased their body weight along the protocol similarly and reached equal values at the end of the experimental protocol, as shown in Supplementary Table S2. Training significantly increased physical capacity of SHR groups, compared with their respective sedentary groups, which was not affected by pharmacological treatment. All SHR groups had similar TA and LV muscle weight. Supplementary Table S2 shows that sedentary control SHR exhibited a significant higher BP when compared with Wistar sedentary rats (found in Supplementary Table S1). SBP (148 ± 6 vs. 114 ± 4 mmHg, *p* < 0.05), DBP (128 ± 3 vs. 96 ± 2 mmHg, *p* < 0.05) and MBP (135 ± 6 vs. 102 ± 6 mmHg, *p* < 0.05). Captopril, perindopril and aerobic exercise training significantly reduced BP in sedentary SHR, as shown in Supplementary Table S2. Neither captopril nor perindopril induced additional reductions on BP of trained SHR, as shown in Table 2S.

**FIGURE 1:**
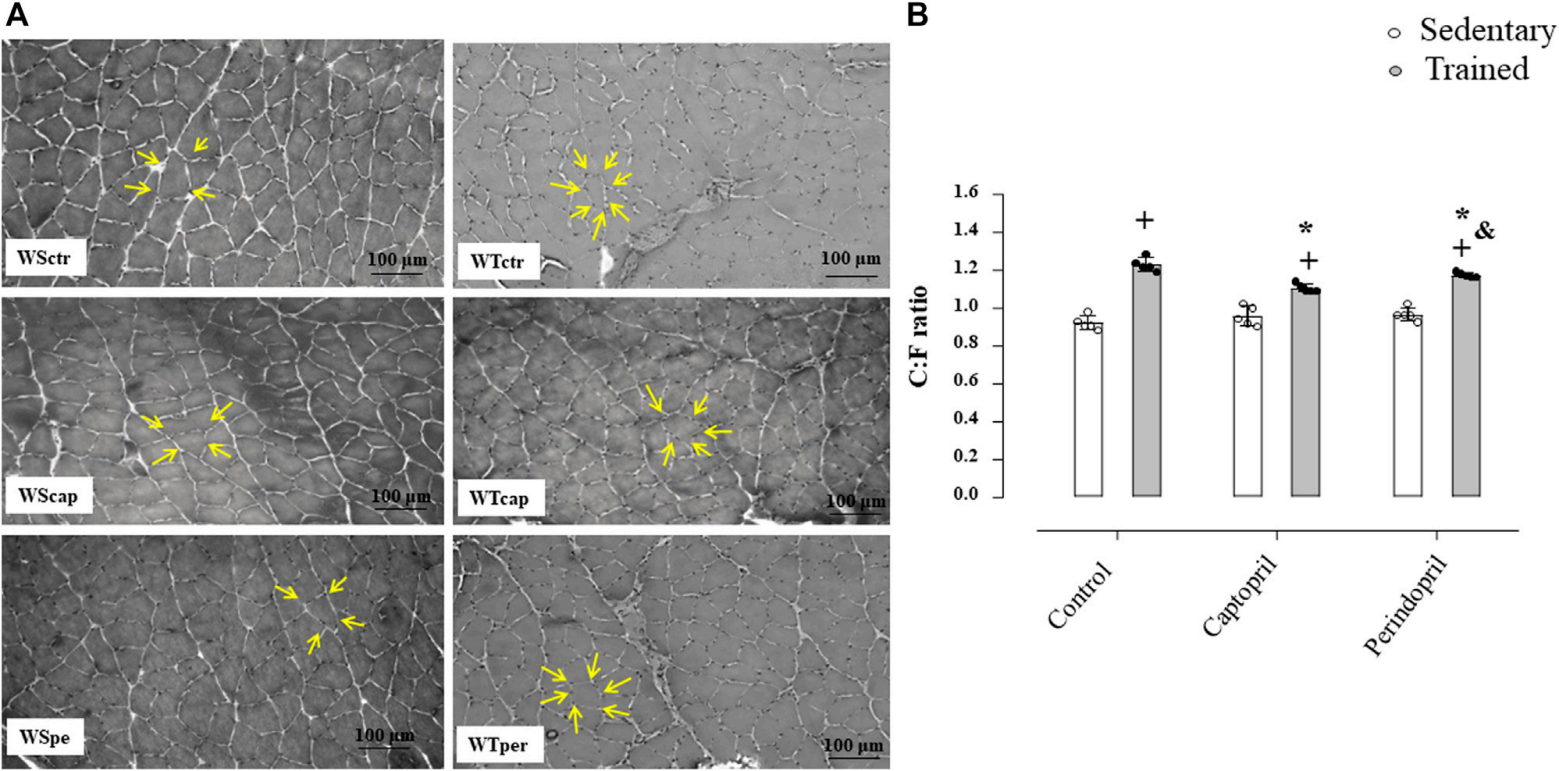
**(A)** Histological sections from the tibialis anterior muscle (TA) of 1 animal from each group stained with hematoxylin and eosin. Bar: 100 µm. **(B)** Densitometric analysis of tibialis anterior muscle capillary-to-fiber ratio (C:F ratio) in all groups: control, captopril and perindopril, in sedentary and trained normotensive rats. Number of rats in each group: 5-6 animals. Significance: + vs*.* sedentary, *vs*.* control, & vs*.* captopril, *p* < 0.05.

Figure 1 A represents histological transverse sections (10 µm) of the tibialis anterior muscle (1 image of each group) stained with hematoxylin and eosin (HE), showing the capillaries around the fibers—yellow arrows (bars: 100µm, 200x) in Wistar rats; B/morphometric analyses of the capillary-to-fiber ratio (C:F ratio) in tibialis anterior muscle of Wistar rats. Figure 1B shows that neither Cap nor Per significantly changed C:F ratio in sedentary Wistar rats. On the other hand, training increased C:F ratio in all groups (+34%, +15% and +21%, for WTctr, WTcap and WTper, respectively, *p* < 0.05), compared with their respective sedentary group in TA muscle. However, captopril-trained rats presented C:F ratio 10% lower than control-trained rats (*p* < 0.05.) while perindopril-trained rats had C:F ratio 4% lower than respective control (*p* < 0.05.). Therefore, perindopril-trained group had C:F ratio 6% higher than captopril-trained group (*p* < 0.05).

**FIGURE 2:**
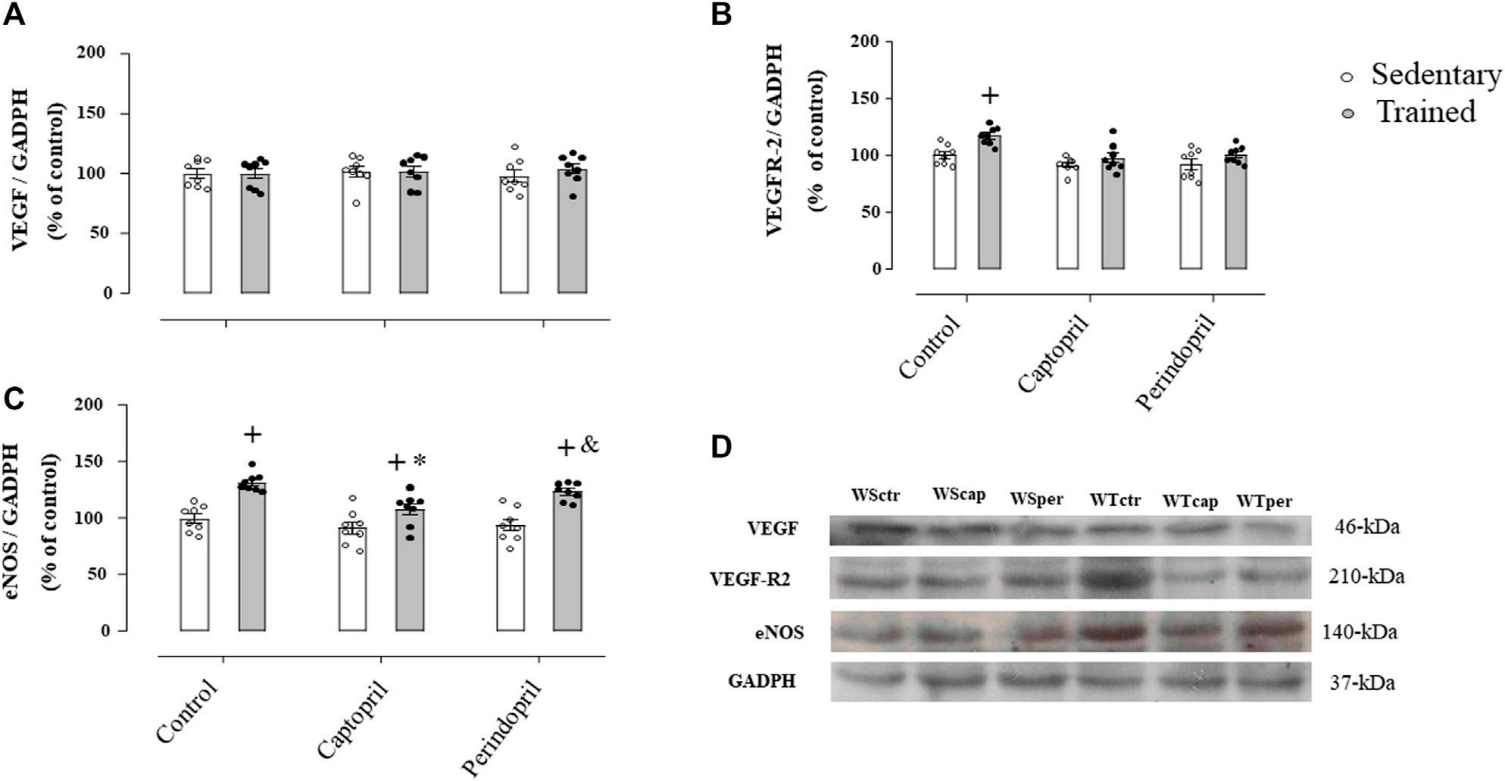
Values of densitometry analysis of VEGF **(A)**, VEGFR-2 **(B)** and eNOS **(C)** protein level normalized by GAPDH in tibialis anterior muscle (TA) in all groups: control, captopril and perindopril, in sedentary and trained normotensive rats. **(D)** Western blot of vascular endothelial growth factor (VEGF165), vascular endothelial growth factor receptor-2 (VEGFR-2), endothelial nitric oxide synthase (eNOS) and glyceraldehyde-3-phosphate dehydrogenase (GAPDH) in TA muscle. For each sample, 50 µg of total protein was loaded. Number of rats in each group: 7-8 animals. Significance: *+* vs*.* sedentary, * vs*.* control, & vs*.* captopril, *p* < 0.05.

Figure 2 shows the protein level of VEGF (panel A), VEGFR-2 (panel B) and eNOS (panel C) analyzed in all groups of normotensive rats. As shown, VEGF protein level was not changed neither by training nor ACE inhibitor treatments. On the other hand, training significantly increased VEGFR-2 protein level in control group (+17%, *p* < 0.05, Figure 2B), which was completely inhibited by captopril or perindopril (Figure 2B). Trained groups presented higher eNOS protein level (panel C) (+32%, +17%, +32%, for WTctr, WTcap and WTper, respectively, *p* < 0.05), compared with their respective sedentary groups. As demonstrated in Figure 2C, WTcap group had lower eNOS protein level compared with control (−8%, *p* < 0.05.), but perindopril did not affect the increase in eNOS protein level induced by training, since WTper group had similar results compared with control-trained Wistar rats. Figure 2D also shows the representative gel of each group for all proteins: VEGF, VEGF-R2, eNOS and GAPDH.

**FIGURE 3:**
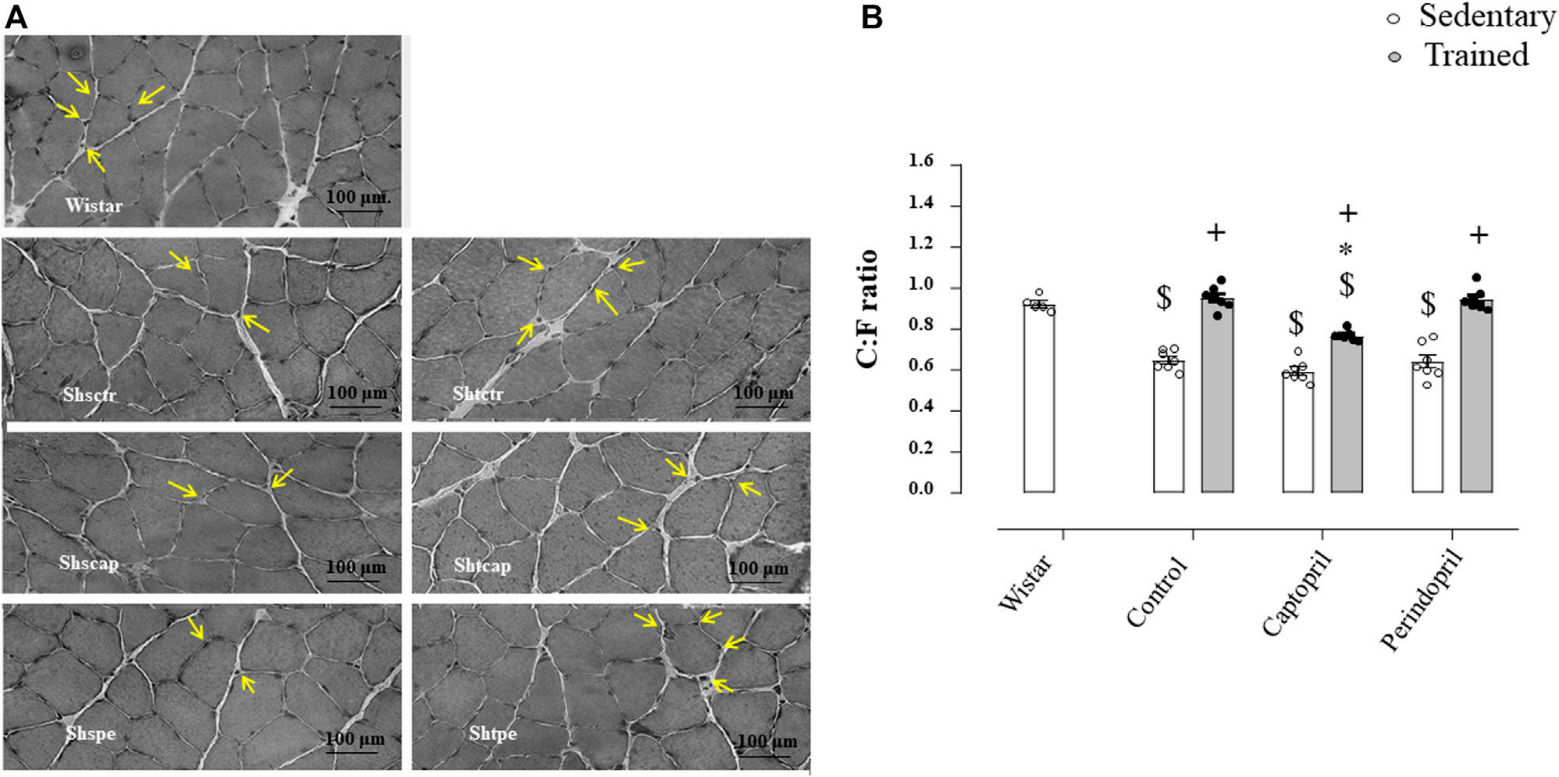
**(A)** Histological sections from the tibialis anterior muscle (TA) of 1 animal from each group stained with hematoxylin and eosin. Bar: 100 µm. **(B)** Densitometric analysis of tibialis anterior muscle capillary-to-fiber ratio (C:F ratio) in all SHR groups: control, captopril and perindopril, in sedentary and trained SHR. Number of rats used: 5-7 each group. Significance: $ vs. wistar, * vs. control, + vs. sedentary, *p* < 0.05.

Figure 3 A represents histological transverse sections (10 µm) of the tibialis anterior muscle (1 image of each group) stained with hematoxylin and eosin (HE), showing the capillaries around the fibers—yellow arrows (bars: 100 µm, 400x) in SHR; B/morphometric analyses of the capillary-to-fiber ratio (C:F ratio) in tibialis anterior muscle in SHR. Sedentary SHR had lower C:F ratio compared with Wistar rats (−30%, −36% and −31%, for SHRSctr, SHRScap and SHRSper, respectively, *p* < 0.05). Trained SHR had higher C:F in all groups (+47%, + 29% and 48%, for SHRTctr, SHRTcap and SHRTper, respectively, *p* < 0.05) compared with their respective sedentary groups. SHRTcap presented lower C:F ratio compared with SHRTctr, while SHRTper presented similar values of SHRTctr.

**FIGURE 4:**
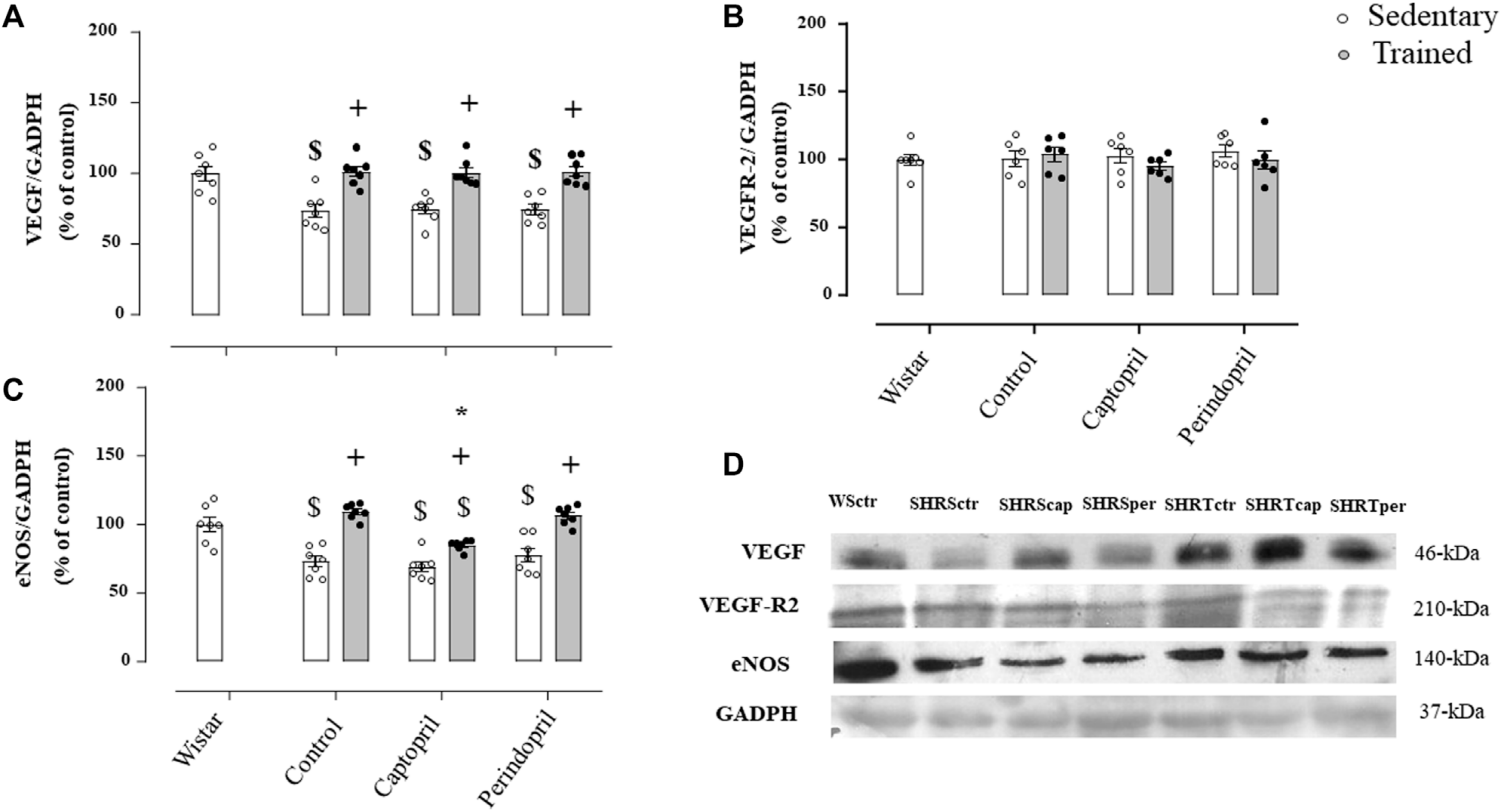
Values of densitometry analysis of VEGF **(A)**, VEGFR-2 **(B)** and eNOS **(C)** protein level normalized by GAPDH in tibialis anterior muscle (TA) of all SHR groups: control, captopril and perindopril, in sedentary and trained SHR. Wistar sedentary group was used as control. **(D)** Western blot of vascular endothelial growth factor (VEGF165), vascular endothelial growth factor receptor-2 (VEGFR-2), endothelial nitric oxide synthase (eNOS) and glyceraldehyde-3-phosphate dehydrogenase (GAPDH) in myocardium muscle. For each sample, 50 µg of total protein was loaded. Number of rats used: 6-7 each group. Significance: $ vs. wistar + vs. sedentary, * vs. control, *p* < 0.05.

Figure 4 demonstrates results of densitometric analysis of VEGF (panel A), VEGFR-2 (panel B) and eNOS (panel C) in SHR groups. The VEGF protein level was significantly reduced in all sedentary SHR groups (−27%, −26% and −26%, for SHRSctr, SHRScap and SHRSper, respectively, *p* < 0.05). Training increased VEGF protein level in all trained hypertensive groups (+38%, + 34% and + 36%, for SHRTctr, SHRTcap and SHRTper, respectively, *p* < 0.05) compared to their respective sedentary hypertensive groups (*p* < 0.05). Figure 4B illustrates that VEGFR-2 protein level was similar in all SHR groups. The eNOS protein level (Figure 4C) was also lower in all sedentary SHR groups (−27%, −31%, −23%, for SHRSctr, SHRScap and SHRSper, respectively, *p* < 0.05), when compared with sedentary Wistar group (*p* < 0.05). Training increased eNOS protein level in all SHR (+50% + 23% and +38%, for SHRTctr, SHRTcap and SHRTper, respectively, *p* < 0.05), compared with their respective sedentary SHR groups. However, trained SHR and treated with captopril showed a significantly lower increase in eNOS protein level (−27%) compared with the SHR control or perindopril treated SHR.

**FIGURE 5:**
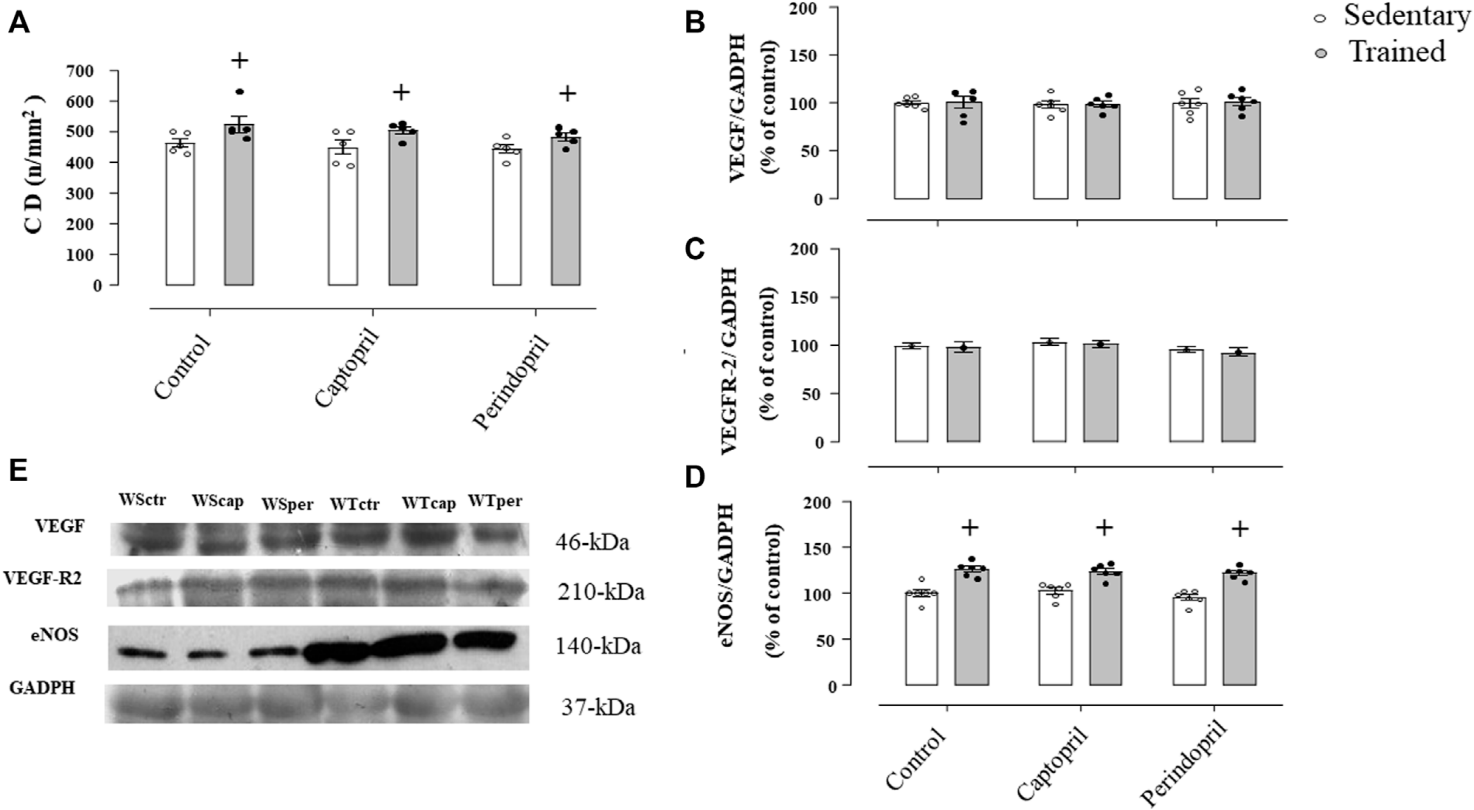
Myocardium capillary density CD, **(A)** in all Wistar groups analyzed: control, captopril and perindopril, in sedentary and trained normotensive rats. Number of rats in each group: 5-6 animals. Densitometric analysis of the VEGF **(B)**, VEGFR-2 **(C)** and eNOS **(D)** protein level normalized by GAPDH in all groups: control, captopril, and perindopril, in sedentary and trained normotensive rats. **(E)** Western blot of vascular endothelial growth factor (VEGF165), vascular endothelial growth factor receptor-2 (VEGFR-2), endothelial nitric oxide synthase (eNOS) and glyceraldehyde-3-phosphate dehydrogenase (GAPDH) in myocardium muscle. For each sample, 30 µg of total protein was loaded. Number of rats in each group: 7-8 animals. Significance: + vs. sedentary, *p* < 0.05.

Figure 5A shows the myocardium capillaries in Wistar rats. It is possible to observe that neither captopril nor perindopril changed myocardium capillary density in Wistar sedentary rats. However, T increased myocardium CD (+15%, 15% e 12%, for WTctr, WTcap and WTper, respectively, *p* < 0.05) compared with sedentary. Results of densitometric analyses of VEGF (Figure 5B), VEGFR-2 (Figure 5C) and eNOS (Figure 5D) protein level are also shown. ACE inhibitor treatments did not change VEGF or VEGFR-2 protein level after training. Figure 5C demonstrated that eNOS protein level increased after training and captopril or perindopril did not alter this response (+26%, +21% e +28%, for WTctr, WTcap and WTper, respectively, *p* < 0.05).

**FIGURE 6:**
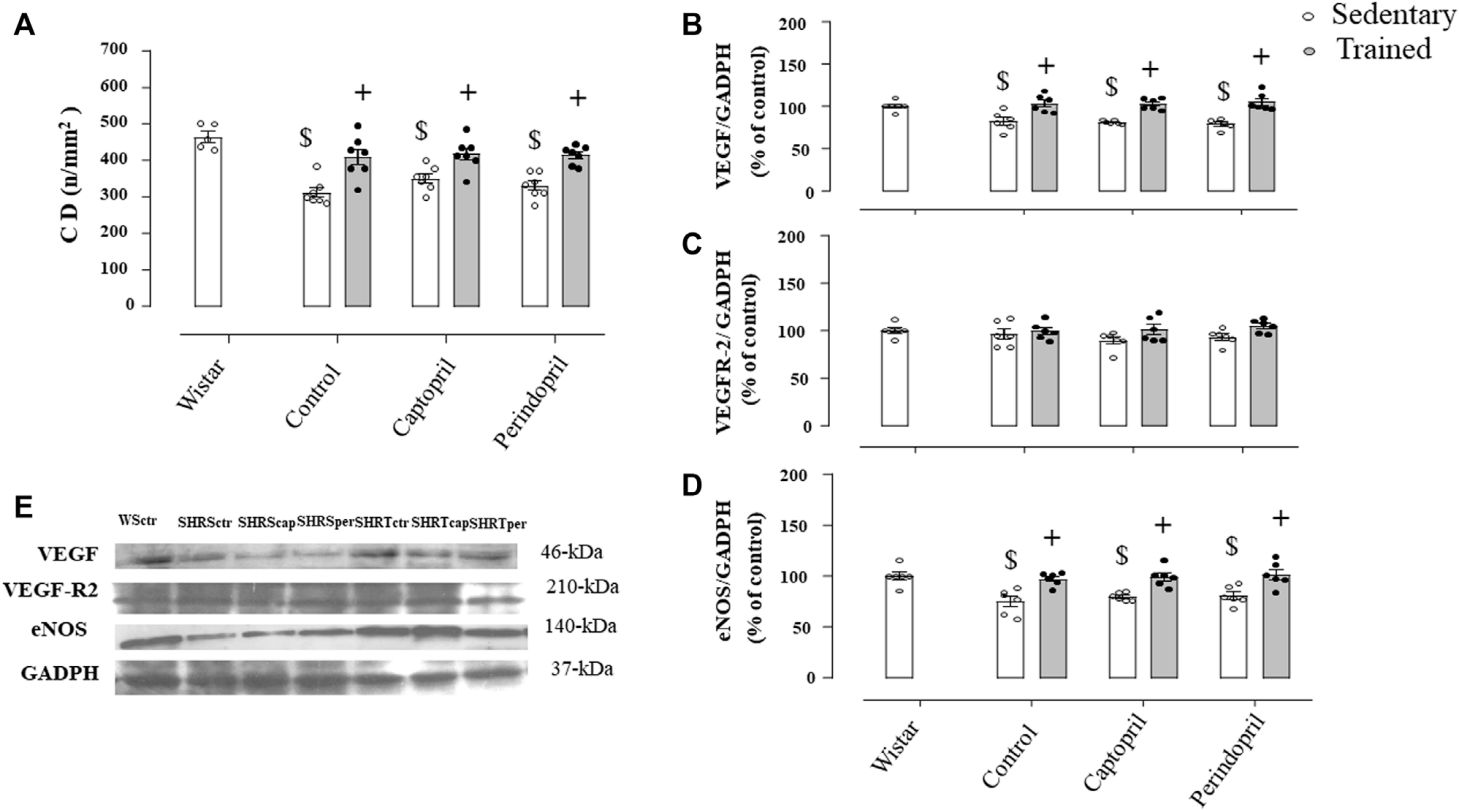
Myocardium capillary density CD, **(A)** in all SHR groups: control, captopril and perindopril, in sedentary and trained SHR. Wistar sedentary group was used as control. Number of rats used: 5-7 each group. Wistar sedentary group was used as control. Densitometric analysis of the VEGF **(B)**, VEGFR-2 **(C)** and eNOS **(D)** protein level normalized by GAPDH in all SHR groups: control, captopril, and perindopril, in sedentary and trained SHR. **(E)** Western blot of vascular endothelial growth factor (VEGF165), vascular endothelial growth factor receptor-2 (VEGFR-2), endothelial nitric oxide synthase (eNOS) and glyceraldehyde-3-phosphate dehydrogenase (GAPDH) in myocardium muscle. For each sample, 30 µg of total protein was loaded. Number of rats used: 6-7 each group. Significance: $ vs. Wistar, + vs. sedentary, *p* < 0.05.

Figure 6A illustrates the myocardium CD in all SHR groups. Myocardium CD was lower in all sedentary SHR less compared with Wistar rats (−24%, −24% e −23%, for SHRSctr, SHRScap and SHRSper, respectively, *p* < 0.05). Training increased myocardium CD in all SHR groups (29%, +28% e +30%, for SHRTctr, SHRTcap and SHRTper, respectively, *p* < 0.05). Figures 6B and C show that ACE inhibitor treatments did not change myocardial VEGF or VEGFR-2 protein level after training in SHR. On the other hand, training increased myocardium eNOS protein level in all groups of SHR.

## 4 Discussion

The main findings of this study were that aerobic training reduced BP in hypertensive animals and significantly increased vessel density in the skeletal and cardiac muscles of normotensive and hypertensive rats. Furthermore, chronic treatment with captopril attenuated training-induced angiogenesis. On the other hand, angiogenesis was less attenuated after perindopril treatment in SHR and this response seems to depend on eNOS production.

The effects of captopril or perindopril on muscle mass, body weight and physical capacity were evaluated in the present study and it was demonstrated that these drugs did not affect the effectiveness of training in improving the physical capacity of the animals, nor in the gain of body weight during the training period, in agreement with other studies (Minami et al., 2007; Guo et al., 2010). In addition, this present study confirmed that training significantly reduced BP of SHR (−28%). Although the mechanisms for BP reduction were not investigated in this present study, it may be related to changes in cardiac output or peripheral vascular resistance, which can be induced by reduced RAS activity followed by lower wall/lumen ratio of arterioles (Amaral and Michelini, 2011; Fernandes et al., 2012a; Duchatsch et al., 2021; Miotto et al., 2021).

In fact, the increase in vessel density induced by training has been associated with BP reduction in SHR (Amaral et al., 2000; Amaral and Michelini, 2011). Both captopril (27%) and perindopril (32%) significantly reduced BP of sedentary SHR as expected (Barroso et al., 2021; Heidari et al., 2022) and the combination of training and pharmacological treatment did not further reduce BP, confirming our previous results shown in SHR (Miotto et al., 2021). Note that neither training not treatment changed BP in normotensive rats.

It is well known that acute or short-term aerobic exercise training induces skeletal muscle capillary growth, as shown in experimental studies (Amaral et al., 2008; Guo et al., 2010; Fernandes et al., 2012b) and the mechanisms responsible for this response involves VEGF signaling pathway and its regulators, such as NO, hypoxia-inducible factor-1 subunit α (HIF-1), peroxisome proliferator-activated receptor- γ coactivator; (PGC-1) (Zmudzka et al., 2022) and RAS (Amaral et al., 2001a). In agreement, the results of this present study showed that sedentary SHR presented lower vessel density compared with Wistar rats, also called rarefaction and, interesting, this response was accompanied by lower levels of VEGF and eNOS, suggesting that these factors are involved also in the maintenance of the microcirculation.

The role of RAS in the physiological angiogenic response has been shown by our group, for instance, treatment with either captopril or losartan blocks the capillary growth induced by 3 days exercise on a treadmill in TA or gastrocnemius muscles (Amaral et al., 2001b). Similarly, lisinopril or losartan blocked the angiogenesis induced by electrical stimulation on TA (Amaral et al., 2001a). Based on these previous results we suggested that angiogenesis was controlled by Ang II levels, since ACE inhibitors blocked the capillary growth induced by exercise or electrical stimulation. In addition, we were concerned about the lack of vessel growth response in skeletal muscle, which would be detrimental to exercisers and users of RAS inhibitors. The results of the present study revealed that 60 days of aerobic exercise significantly increased TA vessel density in all groups, normotensive and hypertensive rats and, chronic treatment with captopril only mitigated this response in Wistar rats and SHR, compared with control rats, unlike the results already observed by our group using 7 days of electrical stimulation or 3 days exercise on a treadmill, which are shorter periods compared with this present study (Amaral et al., 2001a; Amaral et al., 2001b). The new result observed in this present study was that when perindopril was compared with captopril, the results revealed that angiogenesis attenuation induced by perindopril was lower compared with captopril in TA muscle in Wistar rats, but not in SHR, suggesting an advantage of perindopril over captopril in preserving the process of vessel growth after exercise training in both normotensive and hypertensive rats, and this response was greater in SHR. It has been shown that captopril contains the sulfhydryl group, while perindopril contains the carboxyl group. Captopril and perindopril have the same lipophilicity. However, perindopril is a prodrug, which determines higher plasma concentration and plasma half-life, compared to the sulfhydryl group, administered as active drug. Perindopril, a representative of the carboxyl group, reaches plasma concentration in 3–7 h and a plasma half-life of 24 h, while captopril, belonging to the sulfhydryl group, has a plasma concentration of 2 h and a plasma half-life of 3–4 h, therefore, the effects of ACE inhibitors on the carboxyl group seem to be more prolonged than on the sulfhydryl group. In addition, it was shown that ACE inhibitors such as enalapril, perindopril and ramipril blocked 100% of plasma ACE activity in normotensive animals, on the other hand this blockade with captopril was 83%. Thus, the inhibition of plasma ACE activity by captopril is lower compared to perindopril (see Ferrari et al. to review about different ACE inhibitors) (Ferrari et al., 2005). Consequently, less attenuation of ACE activity (by captopril vs. perindopril) results in greater degradation of circulating bradykinin and less interaction with its B2 receptor. In fact, lower concentrations of circulating bradykinin and lower interaction with B2 receptors would promote lower eNOS protein production in skeletal muscle, as demonstrated in the results of the present study. In this present study we used the doses of captopril and perindopril recommended to treat hypertension (Sattar et al., 1985; Minami et al., 2007; Barroso et al., 2021).

This response was independent of the blood pressure values, which was similar among groups after training or treatments. In agreement, Silva Jr. et al. (Silva et al., 2017) have shown that aerobic exercise reduces plasma Ang II, increases Ang (1-7) and decreases AngII/Ang (1-7) ratio in both normotensive and hypertensive rats. Guo et al. (Guo et al., 2010) have also demonstrated similar results after perindopril treatment and exercise training in female Wistar rats, but, as far as we know, this is the first study investigating the effects of two different ACE inhibitors (captopril and perindopril) on skeletal muscle and cardiac angiogenesis induced by aerobic exercise training in normotensive and hypertensive rats. Furthermore, it was not known whether the same aerobic training protocol (same intensity, volume, and frequency) would achieve the same effects on skeletal and cardiac muscle tissue.

Unlike skeletal muscle, training-induced myocardial angiogenesis was not affected by these ACE inhibitors, which was a major finding. In SHR, aerobic exercise training reversed the myocardial capillary rarefaction, indeed. The lack of inhibition of the angiogenesis by captopril in the myocardium may be interpreted as a paradoxical finding since we and others have shown that angiotensin II is a recognized angiogenic factor (Munzenmaier and Greene, 1996; Greene and Amaral, 2002). However, findings in the literature (Kalkman et al., 1999) have also shown that captopril did not affect vessel growth in the myocardium of infarcted rat models. In addition, both ACEIs did not reduce protein production or e-NOS gene expression in muscle tissue. The mechanisms for this different response are not completely understood, but a potentiation of kinins, through an angiotensin converting enzyme inhibitor-related reduction in their breakdown, could increase vascularization. In addition, the number of bradykinin receptors may vary between tissues. Yazawa et al. (2011) have also shown that perindopril treatment promoted myocardial capillary formation as well as attenuated cardiac remodeling in Dahl-sensitive rats fed a high-salt diet and these authors suggested that this benefic cardiac effect was associated with activation of the bradykinin-nitric oxide pathway in the heart. In addition, another possible explanation for this response could be the activation of the counter-regulatory RAS-ACE2-Ang-(1–7)-Mas receptor axis, which may be induced by exercise training (Nunes-Silva et al., 2017; Silva et al., 2017; Frantz et al., 2018). It has been shown that aerobic physical exercise increases angiotensin (1-7) and its receptor-MAS on the heart of SHR (Filho et al., 2008) and Ang (1-7) promotes cardiac angiogenesis via stimulating the expression of cardiac VEGF-D and matrix metalloproteinase-9 (Zhao et al., 2015).

To understand the contribution of the different ACE inhibitors on angiogenesis induced by aerobic exercise training, we evaluated the protein level of VEGF, VEGFR-2 and eNOS, since they are the most important factors that control capillary growth under several physiological and pathological conditions, as stated before (Milkiewicz et al., 2005; Ferrara, 2009; Gavin et al., 2015). Actually, VEGF and eNOS have reciprocal effects: while VEGF increases phospholipase-C, mobilizes intracellular Ca^++^ and stimulates NO production (Koch and Claesson-Welsh, 2012), NO may stimulates VEGF expression (Milkiewicz et al., 2005). The crucial role of VEGF on skeletal muscle angiogenesis has been confirmed by us and others, using anti-VEGF antibody or genetic manipulation, such as VEGF-knockout mice (Amaral et al., 2001a; Amaral et al., 2001b; Olfert et al., 2009; Delavar et al., 2014). The results of the present study showed that TA or myocardium VEGF protein levels were not changed by training or chronic use of ACE inhibitors in normotensive rats. The lack of VEGF level increase after training, even in a presence of higher capillary density, was already demonstrated by our group and others (Richardson et al., 2000; Amaral et al., 2008; Malek et al., 2010) and this response suggests that high VEGF protein level is necessary to trigger vessel growth process, but when vessel density is already enough, VEGF protein level comes back to basal level. Even for acute exercise, the increase of VEGF is lower in trained individuals compared with sedentary individuals (Richardson et al., 2000). On the other hand, increases in the VEGF protein level, induced by training in this present study, normalized the lower capillary density observed in TA and myocardium of SHR.

While VEGF protein level was not altered after training in Wistar rats, VEGFR-2 protein level was increased after training in control animals, at least in TA muscle, as previously shown in skeletal muscle (Fernandes et al., 2012a; Fernandes et al., 2012b). This response suggests that VEGF pathway may be activated after training and stimulates the anti-apoptotic pathway induced by VEGF-R2 (Fernandes et al., 2012a). In contrast, VEGFR-2 protein level was not changed after training in control SHR. Despite the angiogenic response observed in the myocardium of Wistar rats, the protein level of VEGF and VEGFR-2 did not change.

On the other hand, ACE inhibitors blocked the increases in VEFGR-2 protein level, which could explain part of the attenuated vessel growth in TA found in Wistar and SHR treated groups. These results are in accordance with previous results showing that captopril inhibits the expression of VEGF-R2 (flk-1) in trained animals (Gavin et al., 2000). Although the increase in VEGF and VEFGR-2 protein level was blocked (Wistar) or attenuated (SHR) by both ACE inhibitors, angiogenesis was only attenuated. Therefore, we analyzed eNOS protein level. Several studies have shown that chronic exercise upregulates eNOS mRNA and protein (Fernandes et al., 2012a; Fernandes et al., 2012b) and it has been shown that NO plays an important role in angiogenesis induced by exercise, mainly because NO production is an important signal for VEGF (Morbidelli et al., 1996). Additionally, NO may be stimulated by activation of VEGF-R2 (Koch and Claesson-Welsh, 2012), by shear stress (Hudlicka et al., 2006) and by bradykinin pathway (Silvestre et al., 2001).

In agreement with other studies (Fernandes et al., 2012a; Fernandes et al., 2012b), the results of the present study revealed that aerobic exercise training increased skeletal muscle eNOS protein level in all trained rats, Wistar and SHR, however, this increase was attenuated only by captopril treatment, compared with control and perindopril-treated rats.

This response may be explained because these ACE inhibitors differ in the chemical structure, potency, bioavailability, plasma half-life, route of elimination, distribution and affinity for tissue-bound ACE (Brown and Vaughan, 1998). Accordingly, perindopril, unlike captopril, lisinopril, and enalapril, is a new long-acting prodrug ester, non-sulfhydryl-compound that inhibits 50% of ACE activity at a lower concentration than enalapril (Louis et al., 1992). Perini et al. (2020) published a recent review and demonstrated that perindopril inhibits ACE activity with higher potency that captopril or lisinopril. In agreement, Campbell et al. (1994) have shown that lisinopril (sulfhydryl-compound like captopril) had about one-10th the potency of perindopril with respect to its effects on plasma angiotensin peptide levels. Also, since perindopril has high lipid solubility, it crosses the brain–blood barrier and decreases brain ACE activity by 50%, different from enalapril. In addition, perindopril has higher affinity to bradykinin than other ACE inhibitors (Ceconi et al., 2007). Bradykinin is involved in the regulation of angiogenesis through a Ca^2+^- mediated mechanism. Indeed, bradykinin binds its B2 receptor and activates the phospholipase-C/IP-3/DAG pathway, which mobilizes Ca^2+^ ions and stimulates eNOS (Valdes et al., 2008). In this sense, some authors have confirmed that bradykinin plays an important role in angiogenesis via the B2 receptor in ischemia models, mediated by eNOS (Emanueli et al., 2001).

Taken together all this information, we can propose that, since VEGF and VEGFR-2 protein levels were similar in Wistar rats, the captopril-treated group had less exercise-induced capillarization due to its lower eNOS protein level when compared with rats treated with perindopril. Likewise, the lower increase in eNOS protein level induced by exercise in SHR was the main responsible for the lower capillarization found in captopril-treated rats, since the increase in VEGF was similar in all SHR groups. Although the increase in TA capillary density was differently modulated by captopril and perindopril, cardiac angiogenesis was preserved in trained and treated-rats and this response was modulated by eNOS protein level in both Wistar and SHR. It is important to highlight that these effects were found in rats trained for 5 days/week and 1 h/day (300 min/week) and it is not known whether lower training volumes would achieve the same results. In addition, it is essential to be aware that pharmacological treatment can cause several side effects beyond the attenuation of vessel growth and the combination of antihypertensive drugs and exercise may have different effects influenced by the genetic background and nutritional condition. These aspects encourage the continuation of studies in this area, seeking personalized treatment, with the aim of achieving a better quality of life.

In conclusion, considering only the aspect of vessel growth, since both pharmacological treatments reduced BP in SHR, the result of the present study suggests that perindopril could be a drug of choice over captopril for hypertensive practitioners of aerobic physical exercises, especially considering that it does not attenuate angiogenesis induced by aerobic physical training in skeletal and cardiac muscles.

## Figures and Tables

**Table:** 

**PVR**	Peripheral vascular resistance
**ACE**	Angiotensin converting enzyme
**ANOVA**	Analysis of variance
**BP**	Blood pressure
**Ang II**	Angiotensin II
**BW**	Body weight
**CD**	Capillary density
**C:F Ratio**	Capillary-to-fibers ratio
**DBP**	Diastolic blood pressure
**eNOS**	Endothelial nitric oxide synthase
**GAPDH**	Glyceraldehyde 3-phosphate dehydrogenase
**HR**	Heart rate
**MBP**	Mean blood pressure
**NO**	Nitric oxide
**T**	Physical training
**RAS**	Renin angiotensin system
**SBP**	Systolic blood pressure
**SHRSctr**	Sedentary hypertensive control
**SHRScap**	Sedentary hypertensive and treated with captopril
**SHRSper**	Sedentary hypertensive and treated with perindopril
**SHRTctr**	Control trained hypertensive
**SHRTcap**	Hypertension trained and treated with captopril
**SHRTper**	Hypertension trained and treated with perindopril
**WSctr**	Normotensive sedentary control
**WScap**	Sedentary normotensive and treated with captopril
**WSper**	Sedentary normotensive and treated with perindopril
**WTctr**	Normotensive trained control
**WTcap**	Normotensive trained and treated with captopril
**WTper**	Normotensive trained and treated with perindopril
**TBS-T**	Tris-buffered saline with tween- 20
**TA**	Tibialis anterior
**Tmax**	Maximum physical capacity test
**VEGF**	Vascular endothelial growth factor
**VEGFR-2**	Vascular endothelial growth factor receptor 2

## Data Availability

The original contributions presented in the study are included in the article/Supplementary Material, further inquiries can be directed to the corresponding author.
