# Supplementary material for: Exercise-induced angiogenesis is attenuated by captopril but maintained under perindopril treatment in hypertensive rats
Source: Front Physiol. 2023 May 22;14:1147525. doi: 10.3389/fphys.2023.1147525 (PMC10239938; doi:10.3389/fphys.2023.1147525)
Supplement: Supplementary file 1 [file DataSheet1.PDF]

Supplementary Material

Exercise-induced angiogenesis is attenuated by captopril but maintained under perindopril treatment in hypertensive rats.

Anderson G. Macedo[‡1]**, Danyelle S. Miotto**[‡1,2]**, Lidieli P. Tardelli**[2]**, Carlos F. Santos**[3]**, Sandra L. Amaral**[1.2]

*** Correspondence:** Corresponding Author: Sandra Lia Amaral. PhD.
amaral.cardoso@unesp.br

Supplementary Table 1

Table 1S. Values of delta Tmax, final body weight, tibialis anterior (TA) and myocardium muscle mass and hemodynamic values of Wistar rats.

	Delta Tmax (km /h)	Final Body weight (g)	TA (mg/cm)	Heart (mg/cm)	SBP (mmHg)	MBP (mmHg)	DBP (mmHg)
**WSctr**	-40.4 ± 24.2	395.6 ± 10.6	168.2 ± 4	266.1 ± 5	114.1 ± 3.8	102.5 ± 1.7	96.9 ± 2.2
**WTctr**	405.1 ± 43 +	402.8 ± 7	173.2 ± 5	268.4 ± 5	111.5 ± 2.1	103.0 ± 1.8	98.8 ± 1.9
**WScap**	-36.7 ± 17.8	391.5 ± 7.8	173.9 ± 3	263.4 ± 4	117.4 ±2.8	107.8 ± 2.6	103.0 ± 3.1
**WTcap**	387 ± 25.5 +	384.2 ± 8.7	166.5 ± 3	271.4 ± 6	115.2 ±2.6	106.7 ± 2	100.5 ± 1.5
**WSper**	-63.6 ± 26.2	386.8 ± 7.6	173.5 ± 2	258.7 ± 4	110.3 ± 3.7	104.9 ± 3.7	102.2 ± 4.3
**WTper**	396.8 ± 56.5 +	388.6 ± 14.8	177 ± 4	259.6 ± 7	109.5 ± 3.3	103.5 ± 2.4	100.2 ± 1.5

WSctr: normotensive sedentary control; WTctr: normotensive trained control; WScap: sedentary normotensive and treated with captopril; WTcap: normotensive trained and treated with captopril; WSper: sedentary normotensive and treated with perindopril; WTper: normotensive trained and treated with perindopril. Tmax (maximum capacity test); SBP (systolic blood pressure); MBP (mean blood pressure); DBP (diastolic blood pressure). Significance: + vs sedentary; $ vs control; p <0.05.

Supplementary Table 2

Table 2S. Values of delta Tmax, final body weight. tibialis anterior (TA) and myocardium muscle mass and hemodynamic values of hypertensive rats.

	**Delta Tmax (km /h)**	**Final Body weight (g)**	**TA (mg/cm)**	**Heart (mg/cm)**	**SBP (mmHg)**	**MBP (mmHg)**	**DBP (mmHg)**
**SHRSctr**	-48.8 ± 24.7	301.5 ± 9.3	122.9 ± 4.5	282 ± 8.9	148.4 ± 6.4	135.4 ± 5	128.1 ± 3.5
**SHRTctr**	500.7 ± 21 +	304 ± 6.5	122.7 ± 3.3	264.4 ± 12.3	106.2 ± 3.1 +	95.4 ± 4 +	90.7 ± 3.1 +
**SHRScap**	-108.8 ± 20.4	291.1 ± 6.6	120.1 ± 4.6	265.5 ± 12.2	108.2 ± 10 *$*	93.3 ± 10.1 *$*	87.1 ± 10.2 *$*
**SHRTcap**	457.2 ± 30.9 +	297.6 ± 8.6	121.1 ± 4	263.2 ± 6.4	108.4 ± 8.3	91.4 ± 11.1	81.7 ± 11.5
**SHRSper**	-62.3 ± 16.9	294.1 ± 8.8	128 ± 4	270.8 ± 8.7	101.1 ± 2.8 *$*	87.4 ± 4 *$*	79.7 ± 5.8 *$*
**SHRTper**	503 ± 34.9 +	308.7 ± 7.9	128.6 ± 7	261.3 ± 7.1	99.1 ± 3.3	87.1 ± 5	82.59 ± 5.1

SHRSctr: sedentary hypertensive control; SHRTctr: control trained hypertensive; SHRScap: sedentary hypertensive and treated with captopril; SHRTcap: hypertension trained and treated with captopril; SHRSper: sedentary hypertensive and treated with perindopril; SHRTper: hypertension trained and treated with perindopril. Tmax (maximum capacity test); SBP (systolic blood pressure); MBP (mean blood pressure); DBP (diastolic blood pressure). Significance: + vs sedentary; *$* vs control; $p < 0.05$.
